# Preparing parents for parenthood: protocol for a randomized controlled trial of a preventative parenting intervention for expectant parents

**DOI:** 10.1186/s12884-018-1939-2

**Published:** 2018-07-28

**Authors:** Mandy Mihelic, Alina Morawska, Ania Filus

**Affiliations:** 1 Parenting and Family Support Centre, School of Psychology, The University of Queensland Brisbane, St Lucia, QLD 4067 Australia; 2 Center for Self-Report Science, Center for Social & Economic Research, University of Southern California, Los Angeles, USA

**Keywords:** Parenting intervention, Preventative parenting, Infants, Randomised controlled trial

## Abstract

**Background:**

Becoming the parent of a new baby comes with a range of challenges including difficulties with emotional adjustment, couple relationship issues and difficulty managing common infant behaviors, such as crying and sleep problems. This time can be especially challenging for couples who experience a range of risk factors. Previous parenting interventions for parents of babies have shown mixed results. This protocol paper describes a randomized controlled trial of a group-based parenting intervention for high-risk parents expecting their first baby.

**Methods/design:**

Participants will be randomized to either Group Baby Triple P or Care as Usual (CAU). Group Baby Triple P involves 4 × 2 h group sessions delivered during pregnancy and 4 individual telephone sessions of 30 min each in the early postnatal period. Outcomes will be assessed via parent self-report questionnaire, home observations and a baby diary 10 weeks and 6 months post-birth. Primary outcomes will be parental confidence and perceived competence. Secondary outcomes will include parental responsiveness and bonding with the baby, relationship happiness, life satisfaction, depression, anxiety and stress, and infant crying and sleep. Analyses will involve a series of rANOVA and rMANOVAs, t-tests and a multilevel modeling approach.

**Discussion:**

A brief summary, strengths and potential implications are discussed.

**Trial registration:**

Australian New Zealand Clinical Trials Registry: ANZCTR 12613000948796. Registered 27 August, 2013.

## Background

The transition to parenthood comes with a range of challenges. New parents are faced with sleep deprivation, mastering infant caregiving tasks, changes in their relationship and substantial lifestyle changes [1, 2]. Postnatal depression in both mothers and fathers is relatively common and its presence has many short- and long­term adverse effects for children’s cognitive, social and emotional development [3, 4]. A significant proportion of couples feel less satisfied with their relationship after becoming parents and couple conflict often increases [5]. At the same time, these early months of family formation are crucially important for the infant’s mental health and development as well as for the mental wellbeing of parents. It is widely accepted that dysfunctional parenting practices and family conflict are generic risk factors that impact on a child’s development and contribute to the occurrence of emotional and behavioral problems from a very young age [6]. Longitudinal studies have shown that stress and distress in parents during the early years of family formation lead to negative outcomes for children lasting into the preschool and primary school years [7, 8]. Therefore, preparing couples for the transition to parenthood and supporting new parents in their new role is a worthwhile investment.

### Difficulties experienced at the transition to parenthood

Becoming a parent means adjusting to many changes in life and this process of psychological adaptation can place a strain on one’s mental health as well as on one’s personal relationships. It also means managing common, albeit distressing baby behaviors, such as problems with sleeping and crying. Severe sleeping and crying problems affect approximately 20% of infants [9, 10]. Before the birth of their first baby, less than half (44%) of parents report feeling adequately prepared for parenthood. After their child is born, a mere 18% report feeling confident as parents [11]. As many as a third of women report problems caring for themselves and their baby and many women, especially first time mothers view current postnatal services as inadequate [12]. In addition to this lack of confidence, some mothers and fathers experience more severe, negative adjustment difficulties, such as symptoms of depression [up to 22%],[13], lowered self-esteem, anxiety [up to 15%]; [14], feelings of fatigue and extreme exhaustion [15–20].

The couple relationship is equally challenged during this period: numerous studies have shown a decrease in relationship satisfaction alongside a decrease in quality time spent together, positive communication and sexual activity [15, 21]. Low relationship satisfaction is associated with increased conflict, individual psychological distress, negative parent­child relationships and negative child outcomes [22, 23].

### Risk and protective factors at the transition to parenthood

Several identified risk factors at the transition to parenthood are associated with a range of negative outcomes for parents, and subsequently their children. Non-modifiable risk factors include low socio-economic status and unplanned pregnancy [24–27]. A range of modifiable risk factors include prenatal depression and anxiety [28], low relationship satisfaction [22, 23], low social support [29, 30] and low confidence [31].

In particular, anxiety and depression in the prenatal and postnatal period have been associated with adverse outcomes for the child, including child maladjustment and internalising difficulties [28], negative behavioural activity at four months of age [32] and lower infant cognitive and psychomotor development [4]. Therefore, addressing these risk factors early on is crucial in preventing poor child development and behavioural outcomes.

The impact of a negative couple relationship can also lead to detrimental child outcomes. Research suggests that problems in the couple relationship can spill over onto the parent-child relationship [33], which in turn can negatively and indirectly contribute to the child’s development through changes in parenting [34]. For example, couple conflict can influence the child’s mental health through an effect on the child’s emotional security [35].

The parenting aspect itself is also an important contributor to child outcomes. Parental self-efficacy and maternal/paternal responsiveness are two protective factors that have been linked to better outcomes for parents of infants. Parental self-efficacy or confidence is related to more realistic parenting expectations during pregnancy [36], better coping with challenges of the early postnatal period, better couple relationship quality, greater satisfaction with their infants [37], reduced depression [38] and more competent and positive parenting skills [39, 40]. Self-efficacy has also been related to the second protective factor, responsiveness, which refers to positive parental interactive behaviors and sensitivity to infants [41]. Sensitive responsiveness aids in the parents’ ability to understand and respond to infant signals [39] and is an important factor in the development of secure infant attachment [42]. A link to improved infant sleep has also been suggested [43] as well as in reducing infant crying problems [44].

### Previous interventions at the transition to parenthood

The etiology of emotional and behavioral problems in early childhood is complex, and both genetic and environmental factors play a role. However, of all the potentially modifiable risk and protective factors that can affect a child’s development, improving parenting skills and confidence holds the greatest potential in improving health and wellbeing of children as well as the family as a whole [6]. Although we know that the early family context is crucial, parents generally receive little preparation beyond the experience of having been parented themselves. A meta­analytic review of the effects of parenting education with expectant and new parents [45] found that, on average, interventions had small to very small effect sizes on parenting, parental stress, child development and mental health, parental mental health and couple adjustment. Those interventions which were shorter (3–6 months) in duration, which had a postnatal component and which targeted high­risk parents tended to have better outcomes. A likely reason for the small effect sizes were that prevention programs such as parenting programs during pregnancy and the early postnatal period, tend to have smaller effects as some parents are expected to adjust well regardless of attending a program. Other systematic reviews have reported on positive intervention effects regarding enhanced sensitivity and infant attachment security, responsiveness, and increased infant sleep and maternal knowledge; however there is little data on the effects on self-efficacy and competence [46–48].

Other programs at the transition to parenthood, such as the Family Foundations program and Couple CARE for Parents focus on the coparenting relationship [49–51]. Positive results of the Family Foundations program have been found on coparenting outcomes, infant soothability and self-soothing, mental health among mothers, and fewer parent-child dysfunctional interactions [50]. Improvements after the Couple Care for Parents program have been found for the couple relationship and parenting stress levels [52], but no significant results were found in regards to parenting itself. However, both the Couple Care for Parents and the Family Foundations mainly focus on the couple relationship, while the parenting component is minimal.

### The triple P - positive parenting program: Baby triple P

The majority of existing programs at the transition to parenthood focus on one area of adjustment only (parenting aspects, couple adjustment or individual adjustment) even though research on the transition to parenthood has shown that all three aspects are important when it comes to creating positive outcomes for infants [3, 22, 23, 53]. Baby Triple P [54] is a psychological parenting intervention aimed to prepare new parents for a positive transition to parenthood by teaching them skills in the domains of parenting their baby, looking after their own wellbeing, as well as maintaining a positive relationship with their partner. Baby Triple P’s unique aspect is its self-regulatory framework and active skills training to enhance self-efficacy and encourage parents to generalize their learnt skills to when their child is older or to other areas and times of their lives. It is part of the Triple P – Positive Parenting Program system of interventions [6].

An initial feasibility trial conducted by Spry [55] investigated Baby Triple P in a universal population. Using a sample of 129 couples, results showed that while the couples who participated in the intervention were satisfied with the help they received, none of the outcomes measured showed significant differences between the intervention and the care as usual group. The author noted that while she aimed for a representative sample of Australian first-time couples, the sample they obtained was older, more educated and better off financially, as well as better adjusted than the general population, thus ceiling effects were found on all outcome measures at baseline.

Further studies reported high acceptability and feasibility of Baby Triple P among families with premature babies [56], mothers in a psychiatric unit [57], and mothers suffering from postnatal depression [58]. However, Tsivos, Calam [58] did not find any significant effects of the intervention for the mothers in the Baby Triple P group compared to the control group, possibly due to the low sample size (*n* = 27) of this pilot study. Additionally, mothers with clinical depression may need greater psychological support than offered by Baby Triple P.

Other trials include an investigation of Baby Triple P postnatally on mother-infant attachment and bonding [59]. While parents rated the program as highly acceptable and satisfactory, there was no effect of Baby Triple P on parent responsiveness and other outcome measures, such as postnatal depression in mothers or fathers. Reasons for the non-significant results similarly include low power in detecting an effect (*n* = 71) as well as potential ceiling effects given the sample was relatively advantaged in terms of their sociodemographic status.

Even though previous trials on evaluating the efficacy of the Baby Triple P program have not found significant outcomes of the intervention, plausible reasons for the lack of significant findings, including small sample sizes and ceiling effects, call for an additional randomized controlled trial to investigate the effects further. This is especially due to the high feasibility and acceptability reported by parents in previous studies as well as the need for a program that prepares new parents for their parenting journey. Fortunately, the ceiling effects in previous trials might indicate that despite the challenges of transitioning to parenthood, the majority of new parents seem to adjust quite well. However, the transition to parenthood can be particularly challenging for families who are at greater disadvantage than others and already experience a range of risk factors. Consequently, developing and evaluating antenatal education programs for high-risk parents is crucial in order to prevent negative outcomes for parents and infants. Yet, according to Gagnon and Sandall [60], the majority of studies assessing antenatal parenting interventions often include only well-educated mothers in the middle to upper socio-economic strata, who might do well regardless of which, or any preparation classes they attend, consistent with the prior findings relating to Baby Triple P. From a cost-benefit analysis, focusing on a higher risk sample for this current trial of Baby Triple is reasonable.

## Aims and hypotheses

In light of the critical importance of a successful transition to parenthood it is clear that strategies need to be developed to support families and enhance the protective factors for babies to ensure positive developmental outcomes. This is vitally important especially for those families with identified risk factors who may find the transition to parenthood particularly challenging. The proposed research will evaluate the efficacy of a tailored parenting intervention, Baby Triple P that combines parenting information, with the provision of strategies to enhance individual wellbeing and couple adjustment for high­risk families.

The aim of this study is to experimentally test the hypothesis that provision of parenting support at the transition to parenthood through Baby Triple P, will lead to improvements in parental confidence and sense of competence in their abilities as parents. Furthermore, we will test the secondary hypotheses that the program will lead to higher levels of responsiveness and bonding with the baby, higher relationship happiness, higher levels of life satisfaction, fewer instances of depression, anxiety and stress, and infants would show less fussing or crying and fewer sleeping problems. It is hypothesized that these positive treatment effects will be maintained at six-month-follow up.

## Method

### Design

The study is a 2 (Baby Triple P vs CAU) × 3 (time: pre-test, post-test, 6-month-follow up) design with multi-informant assessment. The study will experimentally test the relative impact of Baby Triple P against care as usual in a randomized controlled trial aimed at improving baby and parenting outcomes. The CONSORT guidelines for randomized controlled trials will be used. This protocol also adheres to the SPIRIT guidelines.

### Ethics

Ethical approval has been obtained from the Queensland Children’s Health Services Human Research Ethics Committee (HREC/13/QRBW/177) and the University of Queensland Behavioural and Social Sciences Ethical Review Committee (2013000564). The trial has been registered with the Australian New Zealand Clinical Trials Registry (ACTRN12613000948796).

### Participants

Participants will be approximately 110 couples (110 mothers and 110 fathers) expecting their first child and between 20 and 35 weeks of pregnancy, recruited through primary care settings, maternity hospitals, obstetricians, and through targeted mail-out and media campaigns within the Brisbane and Ipswich area in Queensland, Australia.

#### Inclusion and exclusion criteria

Expectant couples have to meet the following inclusion criteria: (a) experiencing a first pregnancy (between 20 and 35 weeks gestation), (b) in a couple relationship, (c) aged at least 18 years, and d) living in the Brisbane area for the duration of the study. Couples will also have to meet at least two of the following risk factors: i) unplanned pregnancy, ii) low education, iii) low income or experiencing financial strain, iv) low relationship satisfaction, v) low social support, vi) history of maternal/ paternal depression or anxiety, vii) current depression or anxiety, viii) low parenting efficacy, ix) low life satisfaction/ happiness, x) aged between 18 and 21 years.

Parents will be excluded if: a) they have an intellectual disability or severe psychopathology which would impair their understanding of the material presented in the program, b) parents do not read and understand English, c) they have a diagnosed genetic disorder, disability in the baby, or experience major complications during pregnancy d) parents are currently receiving psychological help or counseling. Eligibility to these criteria will be based on mothers’ responses only.

#### Recruitment

Participants will primarily be recruited through antenatal clinics at the Royal Brisbane Woman’s Hospital and the Mater Hospital (Brisbane, Australia). Recruitment brochures and posters will be distributed in antenatal clinics. Potential participants will also be approached during antenatal classes. Registration will be via phone, email or on the study website. Parents will have the opportunity to discuss any questions or concerns regarding participation with one of the research team.

Recruitment brochures and posters will be distributed at GP clinics, obstetrician clinics, baby shops and local libraries across the Greater Brisbane, Ipswich and Logan area. This information will also be available through advertisements posted on baby websites and forums, and social media pages (e.g., Facebook), as well as advertisements in the University of Queensland staff newsletter and articles in local newspapers.

Interested parents will be directed to the study website. Mothers will receive information and consent forms detailing the project, and will have the opportunity to address any concerns or questions with the project coordinator before giving consent, and being directed to the online self-report questionnaire. Eligibility will be based on the mother’s responses only and her response will be used to determine if she is eligible based on the inclusion and exclusion criteria a) to d) outlined above. If eligible based on these criteria, the total number of risk factors (i to x) will be summed and if a minimum of two risk factors are present, the mother will be deemed eligible to take part in the trial. Then the father will be asked to complete the self-report questionnaire as well. Following this, the couple will be enrolled in the study by the study coordinator.

#### Randomization

Participant randomization to either the Baby Triple P or CAU group will be conducted by generating a random allocation sequence, by a researcher not involved in the project, using a computer-based random number generator. A pre-prepared series of sealed opaque envelopes, each labelled with a participant ID number, will conceal group allocation from researchers and participants until after completion of the baseline assessment. Immediately after completion of the baseline questionnaires, the research coordinator will open the envelope, and will notify participants of their condition allocation via phone.

**Fig. 1:**
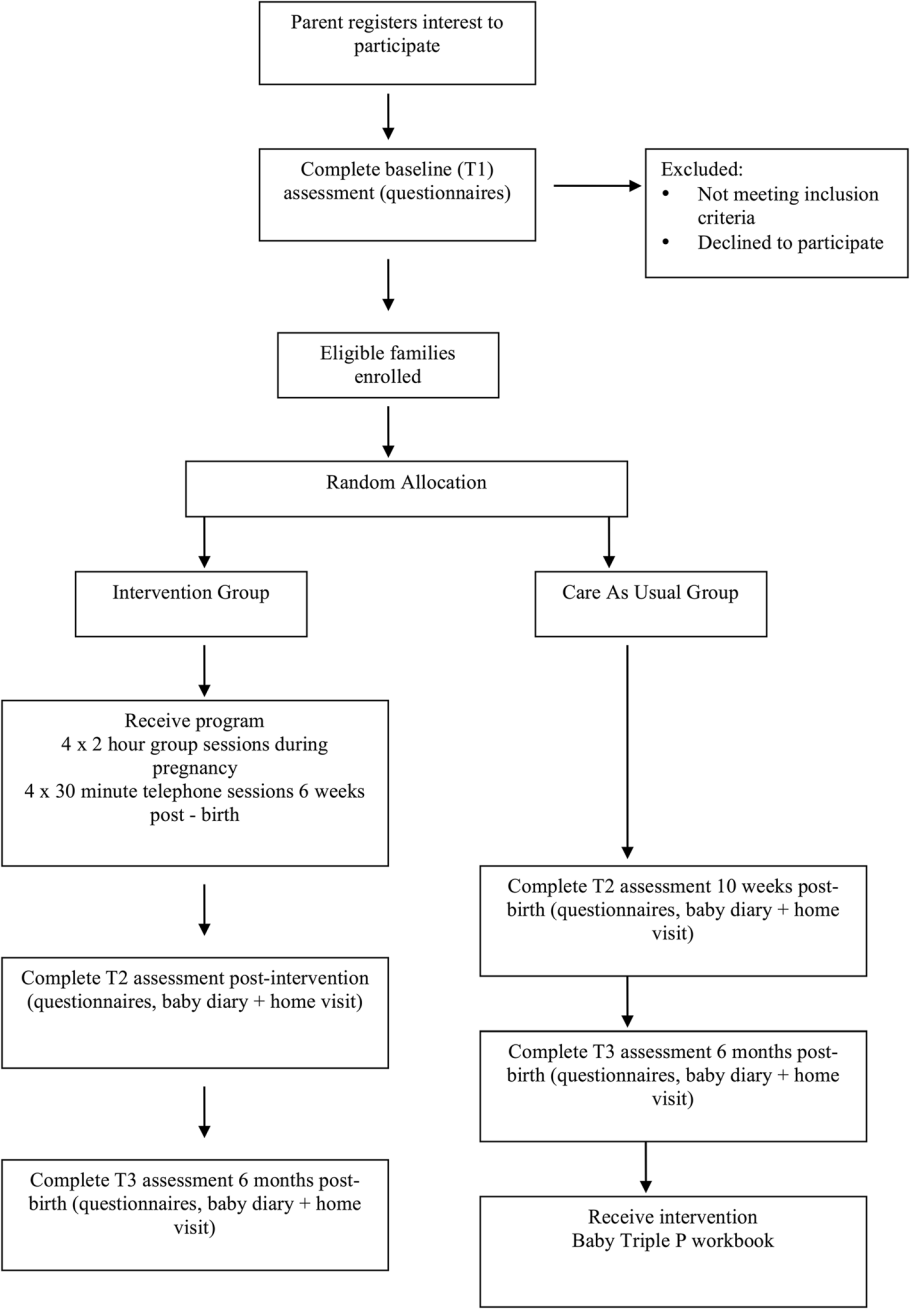
Participant flow through the study

Participants allocated to the intervention condition will attend group sessions based on their preference for day and time, depending on availability of groups. Participants will be allocated to groups as soon as possible after randomization. Figure 1 summarizes the flow of participants through the study.

### Intervention

The intervention will consist of four, 2-h *Baby Triple P* group sessions conducted during pregnancy, followed by four individual 30-min telephone sessions conducted postnatally. The program is based on the same theoretical principles that underpin the Triple P system [6] and sessions are interactive and allow opportunities for discussion.

Baby Triple P is an eight-session psychological intervention, developed by Spry (2013) that aims to prepare couples for a positive transition to parenthood by teaching them core skills in the domains of parenting their baby, taking care of their own wellbeing and maintaining a positive relationship with their partner. The self-regulatory framework will be used to engage participants and facilitate learning. Specifically, it includes active training methods, for example modelling, rehearsal, practice, feedback and goal setting. These techniques are aimed at teaching specific skills and promoting parental competence. The intervention was developed on the basis of a review of the scientific literature outlining the key risk and protective factors for both parents and infants and includes teaching parenting strategies (Sessions 1 & 2), individual coping skills (Session 3) and partner support skills (Session 4).

The parenting strategies are designed to promote a warm and responsive relationship between parents and their babies as well as promoting a positive learning environment for the infant. These first four sessions are conducted in a group setting. Specific parenting strategies covered include spending quality time, affection, settling strategies and providing a stimulating and engaging environment. Individual coping skills taught include acceptance, identifying the relationship between thoughts and emotions, using coping statements, abdominal breathing, being involved in pleasant activities, and developing coping plans. As part of the partner support skills session, couples learn positive speaking and listening skills, casual conversations, communicating their emotional experience and spending quality time together. Throughout the group sessions, parents are given information about common experiences of new parents both at an individual as well as on a couple level. This information aims to promote realistic expectations about the changes that come with the transition to parenthood. Parents further learn how to apply parenting strategies appropriately across the different developmental milestones of the infant. During Session 1, parents are provided with a copy of the Baby Triple P workbook which they could take home and review the content in their own time.

The content is followed up with four postnatal individual telephone sessions (Sessions 5 to 8), which employ a self-regulatory model. The telephone sessions review homework from a previous session, focussing on strengths and areas to improve, and setting and monitoring goals for areas of future change. This teaches participants to reflect on their own behavior as well as the impact it has on their baby. Both the prenatal group sessions and the telephone sessions will be delivered by the first author and trained interns in clinical psychology.

### Care as usual

Couples in CAU will complete assessments at baseline, ten weeks post birth, and at six months post birth. These are the same time points at which the intervention group complete their assessments. During this time, families will continue to receive regular primary care and other pre and post-natal services, as appropriate, including antenatal classes, which may be offered at the mother’s hospital, as well as other services provided by GPs or obstetricians. These services were also available to mothers in the Baby Triple P group. Other than regarding issues of assessment, these parents will have no contact with the research team, but are free to access support from other community services and will be given appropriate referral information should they ask for assistance. After the 6-month follow-up assessment, families will be offered the Baby Triple P workbook.

### Protocol adherence

Practitioner who deliver the intervention are trained using a standardized system of training and accreditation, designed to promote program use and fidelity. Sessions are delivered according to a standardized manual and practitioners received regular clinical supervision. Each practitioner will complete a protocol adherence checklist for each session conducted, which will be coded by a research assistant who is independent of the delivery of the sessions but familiar with the intervention and protocols for adherence. Using structured session checklists, videotapes of group sessions will be independently coded for protocol adherence. A second rater will code 25% of the sessions to establish inter-rater reliability (kappa).

### Assessment

Table 1 provides an overview of the assessment measures and the time points at which they are assessed. All parent-report measures will be provided in a written (online or printed) self-administered questionnaire format, and will take approximately 30 to 40 min to complete. Most of the measures used have been previously validated and are commonly used. Socioeconomic status (e.g., income, occupation status, parent education), age, history of mental illness and ethnic background will be assessed using the *Family Background Questionnaire* [61]*.* Only the mother will complete this measure, as well as the *Cambridge Worry Scale* [CWS; 68].

**Table 1:** Summary of Assessment Measures

Construct	Assessment Measure	Time Point
Demographics	*Family Background Questionnaire*	T1
Primary Outcomes		
Parental Confidence	*Maternal Self-Report Inventory*	T1, T2, T3
Parental Self-Efficacy	*Maternal Efficacy Questionnaire*	T2, T3
Secondary Outcomes		
Parental Mental Health		
Depression	*Edinburgh Postnatal Depression Inventory*	T1, T2, T3
Anxiety	*Depression Anxiety Stress Scale 21*	T1, T2, T3
Pregnancy Worry (mothers only)	*Cambridge Worry Scale*	T1, T2, T3
Life Satisfaction	*Oxford Happiness Questionnaire*	T1, T2, T3
Expectations	*Prenatal Maternal Expectations Scale*	T1
Family and Social Support		
Couple Relationship	*3 items from PAFAS, 2 items from HCTC & 1 overall item*	T1, T2, T3
Social Support	*Social Support Scale*	T1
Mother-Child Relationship		
Maternal Responsiveness	*Maternal Infant Responsiveness Inventory*	T2, T3
Observed Mother-Infant Responsiveness	*The Care Index*	T2, T3
Mother-Child Bonding	*Postpartum Bonding Instrument*	T2, T3
Baby Outcomes		
Problematic Behaviors	*Baby Behaviour Inventory*	T2, T3
Baby Behaviors	*Baby Diary*	T2, T3
Other		
Parent Involvement	*Attendance at group sessions (intervention only)*	During group sessions
Conflicting Advice & Problem Solving	*6 items*	T1, T2, T3 (problem solving only T2 and T3)
Strategies used	*Strategies Checklist*	T2 (intervention only), T3
Services accessed		T2, T3
Client Satisfaction	*Client Satisfaction Scale*	T2

The CWS will only be completed by the mother because several items focus on the pregnancy and birth, thus this scale would not be appropriate for fathers. Both mother and father will complete all other self-report measures. Most of the validated scales were written for mothers to complete, thus wording will be slightly changed to suit the father’s perspective. Where available, internal consistencies from previous studies for mothers and fathers are reported below. Unless otherwise stated, if only one internal consistency is reported, it would refer to mothers’ data only. The home observation will only be conducted with the mother and the baby.

### Primary outcome measures

#### Parenting confidence and sense of competency

The *Maternal Self-Report Inventory* [62] is an overall measure of self-esteem related to the adaptation of motherhood, which has a total of 100 questions across seven subscales. This study will use only the 8-item *General Ability and Preparedness for Mothering Role* subscale of the 26-item short form of the questionnaire. The 5 point scale ranges from 1 = *completely false* to 5 = *completely true.* This subscale has good internal consistency (α = .88). The full scale has demonstrated acceptable external validity [62].

The *Maternal Self­Efficacy Scale* [MSES; 40] is a 10-item measure capturing parent’s self­efficacy and perceived behavioral competence among mothers of infants in the first year. It has previously been used with clinically depressed and non­depressed mothers of infants aged three to 13 months. Nine of the ten items address mother’s feelings of competence in relation to specific domains of infant care, for example soothing the baby, understanding what the baby wants, amusing the baby, disengaging from the baby, performing daily routine tasks, such as feeding, changing and bathing the baby. A final tenth item taps mother’s global feelings of efficacy in mothering. Responses are recorded on a 4-point scale from 1 = *not good at all* to 4 = *very good.* This subscale has good reliability [α = .86; 40, α = .75 and .84, respectively for fathers and mothers; 58] and demonstrated good concurrent validity to the Parenting Sense of Competence Scale [40].

### Secondary outcomes

#### Parental well-being

The *Edinburgh Postnatal Depression Scale* [EPDS; 63] is a 10-item scale and is widely used to screen for parental depression during pregnancy and post-birth. Expectant mothers and fathers are asked to indicate how often they have experienced symptoms relating to depression and/or anxiety over the previous seven days. The response scale is from 0 = *no, not at all* to 3 = *yes, all the time*. The EPDS has good internal consistency for mothers [α = .87; 63] and fathers [α = .81]; [63]. The scale’s sensitivity in detecting depression has been shown to be between 80 and 100% [64, 65].

The *Depression, Anxiety and Stress Scale 21* [66] is a 21-item measure assessing symptoms of depression, anxiety and stress in adults. This study will only use the anxiety and stress subscales. The 4 point response scale is from 0 = *did not apply to me at all, never*, to 3 = a*pplied to me very much, or most of the time, almost always*. The measure has good reliability for the anxiety (α = .81) and stress (α = .88) subscales for both men and women together. The scale demonstrated good convergent validity with the Mood and Anxiety Symptom Questionnaire 90, the Perceived Stress Scale and the Beck Anxiety Inventory [67].

The *Cambridge Worry Scale* [68] is a 17-item prenatal measure assessing worries and concerns in relation to pregnancy. Items assess concerns about health, worry about labor and birth, worry about the baby and concerns about relationships. The 6 point response scale is from 0 = *not a worry* to 5 = *major worry.* The scale has satisfactory internal consistency (α = .79), and good convergent validity with the trait form of the State-Trait Anxiety Inventory [68].

The *Oxford Happiness Questionnaire* (OHQ; Hills & Argyle, 2002) is an 8-item measure assessing personal happiness, based on the original Oxford Happiness Inventory [69]. The 6 point Likert Scale is from 1 = *strongly disagree* to 6 = *strongly agree*. The scale has shown high internal consistency (α = .91) for both men and women, and good convergent validity with variety of measures related to happiness and satisfaction with life [70].

The *Prenatal Maternal Expectations Scale* [71] has 46 items and assesses prenatal expectations pertaining to the following subdomains: characteristics of the baby and child care; degree of enjoyment anticipated in association with parenting, expected changes in the parent’s significant relationships resulting from becoming a parent, anticipated changes in lifestyle or quality of life associated with the parental role, and projected image of self as a parent. Responses are made on a 5 point scale from 1 = *strongly agree* to 5 = *strongly disagree.* The scale has good reliability (α = .83) and predictive validity [71].

#### Family and social support

The *Social Support Scale* [72] will be used to assess participants’ satisfaction with their social network. Two out of the four items will be used for this study. The first item asks participants how satisfied they are with the extent of formal support (i.e., health professionals) they currently receive. The second item referred asks participants how satisfied they are with the extent of informal support (i.e., provided by family, friends and neighbors) they currently receive (from 1 = *Not at all satisfied* to 5 = *Completely satisfied).*

The *Couple Relationship* will be assessed using three items from the Parenting and Family Adjustment Scale [PAFAS]; [73], two items from the Household and Childcare Task Checklist [74] measuring overall perceived fairness and satisfaction with the division of tasks, and one overall item on global relationship satisfaction from the *Relationship Quality Index* [75]. For the purpose of the current study, the PAFAS items assessing the couple relationship were slightly reworded to suit the context of expecting/ having a baby.

For both the social support and couple relationship measures, only a selected few items were chosen to be included in order to limit the total length of the questionnaires. The social support measure and one item from the couple relationship measure were intended as a risk factor indicator only to be used for edibility assessment, rather than an outcome measure.

#### Mother-child relationship

The *Maternal Infant Responsiveness Instrument* [76] is a 22­item scale designed to measure the parents’ feelings about the infant and an appraisal of the infant’s responses. Specifically, it measures the parent’s recognition of his/her own responses, the parent’s recognition of the infant’s responses to him/her, and any difficulties they notice in responsiveness. Response options are from 1 = *strongly agree* to 5 = *strongly disagree.* Internal consistency of the measure is good [α = .81 and .88 for mothers and fathers respectively; 58, α = .86 for mothers; 75].

The *Postpartum Bonding Instrument* [77] will assess parent­infant bonding difficulties. This 25–item scale asks parents to rate how often a list of positive and negative thoughts/ emotions are true for them, from 1 = *always* to 6 = *never*. The PBI has four subscales, which reflect impaired bonding, rejection and anger, anxiety about care and risk of abuse. Internal consistency is acceptable (α = .76) and the measure demonstrated good convergent and concurrent validity [78].

##### The *Care Index*

A home observation will be carried out with the mother and the baby at two stages; upon completion of the program and six months post birth. This will not be done with fathers, as scheduling these visits with fathers will not be practical given that most will be in full-time employment. It will be carried out within the context of semi­structured play and naturalistic interaction, lasting approximately 15 min. Mothers will be instructed to interact with their baby as they usually would. With the baby beside her, the mother will first be asked to read an information sheet and the Baby Triple P brochure for 2 min. This is followed by a nappy change lasting 3 min. The mother will then move on to free play with the baby, using age appropriate toys brought by the researcher. After 5 min, the researcher will prompt the mother to leave the room briefly for a maximum of 2 min. This can be reduced to 20–30 s if the baby is upset and is not able to settle on his or her own. Lastly, the reunion context will be recorded for 2 min. Observations will be videotaped. Video recording of the mother’s interaction with the baby (i.e., the 5 min component) will be coded using the *Care Index* [79]. The *Care Index* assesses mother-infant interaction from birth to about two years of age based on a short, videotaped play interaction of 3–5 min (from the five minute play time segment). Trained independent coders will conduct this assessment, who will be blind to the study aims. The CARE-Index is a dyadic procedure that assesses adult sensitivity in a dyadic context. The measure assesses mothers on three scales: sensitivity, control and unresponsiveness. There are also four scales for infants: cooperativeness, compulsivity, difficultness, and passivity. Validity has been demonstrated with the Parent-infant Interaction Observation Scale [80]. Reliabilities will be assessed by calculating the inter-rater reliabilities for a random 25% of the coding between the two coders.

#### Baby outcomes

The *Baby Behaviour Inventory* [BBI; 80] measures the extent of a range of behaviors that parents often find challenging during the first twelve months. In addition, it captures the level of parental confidence in dealing with the behavior. Encompassing 14 items, parents indicate how often certain behaviors occur. Parents are then asked to identify whether they have experienced the behavior as a problem with their baby using a yes/no format. If the behavior is rated as a problem, parents are asked to rate their confidence in dealing with the behavior. The overall intensity scale of the BBI was found to have satisfactory internal consistency (α = .76) and has demonstrated construct validity to the EPDS and the MSES [81].

The *Baby Diary* [82] was modelled on other similar diaries used in previous studies. We will ask parents to fill in a diary for a 24­hour period to identify the pattern of feeding, sleeping, crying and contentedness in babies. Parents will mark the dominant behavior using specific symbols for each of the four behaviors for 48 half-hour time periods from 6 am to 6 am the following day. Only one diary will be completed per time point so that either mother or father can complete it or they can complete it together.

#### Additional variables

*Parent Attendance* will be assessed by the group facilitator taking attendance of both mothers and fathers at the group sessions. This will only be assessed for parents in the intervention group. It will be coded as either present or not present for both mothers and fathers.

The *Conflicting Advice Questionnaire* and *Problem Solving* is a 6­item measure that was developed for this study to assess the extent participants received conflicting information or advice from professionals and from family and friends, as well as how they deal with confusion and problems. First, it asks about the extent of confusion in regards to five areas, separately from professionals, and from friends and family: feeding the baby, settling the baby, putting the baby to sleep, bathing the baby and dressing and changing the baby. Then, participants are asked to write about any other topics where they experience confusion. Next, participants are asked about what they do when they are confused or encounter a problem in regards to their baby. They can tick one or more of the following options: seek out additional information from family, seek out additional information from professionals, research the internet, corroborate with partner about the problem and try to think of a solution together, try out different strategies until one works. The final item allows participants to write about any other problem solving strategies they have been using.

##### Strategies checklist

This checklist asks parents to indicate which of the 29 strategies that are taught in the Baby Triple P program they have used since their baby was born. The response options include *not at all, a little bit, sometimes, a lot* and *all the time.* This measure was specifically created for this study.

##### Services accessed

Parents will be asked which services they have accessed since they had their baby. Ten options will be listed and participants are asked to tick the service they used, how many times they used it, what they used the service for and how helpful they found it (*very, somewhat, not helpful*).

The *Client Satisfaction Questionnaire* [55, 83] is an 8­item questionnaire that assesses participant’s perception of the quality of the program received, how well the program addresses their needs as well as their overall satisfaction with the services provided. Only the Baby Triple P group completed this measure.

## Statistical analyses

Prior to conducting the main analyses, data will be screened for distributional assumptions (e.g., univariate and multivariate normality, outliers and multi-collinearity) as well as inter- and intra-measure consistency. Preliminary analyses will also investigate whether the study groups differ on any demographic characteristics at baseline. This will be examined via linear (for continuous outcomes) and logistic (for categorical outcomes) regression models. If baseline group differences are detected, we will investigate and report the extent to which the results from the planned analyses described below are altered, when these differences are statically controlled. For missing data points, an analysis of missing data will be conducted (see paragraph below on missing data) and imputation methods will be considered.

### Primary analyses

The analyses will evaluate the effects of the intervention on parent and baby outcomes, including the primary outcome measures of parenting confidence and sense of competency. These analyses will include self-reports obtained from parents as well as reports on baby behaviors, baby diaries and observational assessment. A multilevel modeling approach will be used to take into account the repeated measurements and thus non-independence of observations [84]. This approach will be used for variables that have three measurement time points (e.g. Couple Relationship and the EPDS). Dummy codes contrasting the groups will serve as fixed effects, allowing for random intercepts and slopes to vary across individuals. Outcome variables with two time points (e.g., MSES, and the Baby Diary) will be analyzed using a series of rANOVAs and rMANOVAs. The primary analyses will be conducted for mother and father data separately to investigate whether the intervention is beneficial for both parents, or has different effects for mothers and fathers. Significant fixed effects contrasting Baby Triple P with CAU will indicate whether the intervention is an improvement over usual care. Separate models will be estimated for each outcome measure using Bonferroni correction to control for inflation of Type 1 error due to multiple comparisons.

### Sample size and power analysis

The required sample size for the study was calculated to assure 80% power to detect a medium effect size (ES) = 0.5 for a mean difference in the variables of interest between the groups (INT vs CAU). Effect sizes for early intervention studies range from small to large on a range of outcomes [48]. Two studies that assessed parental confidence and competence as an outcome of a parenting intervention found a small [85] and a large [43] effect. Given these are our primary outcome measures, a medium effect size was expected in this study. Furthermore, as our study will recruit an at-risk sample, an expected medium effect is a conservative estimate. We performed power analysis using G*Power software [86] for rANOVA, looking at the within-between interactions and allowing for conservative estimate of intra-individual variability (.5). The analyses indicated that a sample size of 80 is sufficient to detect an ES of 0.5 at the significance level of .05 (two-tailed). This is without taking into account the added power accorded by the rich repeated measures in our data. In multilevel models with repeated measures, the sample size is effectively the number of observations (level-1), not number of participants (level-2 units). Assuming a 20% attrition rate (based on estimate within Bakermans-Kranenburg, van Ijzendoorn [46] review), an available sample of 110 families will be sufficient to detect medium sized effects.

### Missing outcome data

The full information maximum likelihood analysis (FIML) approach will be implemented to accommodate for missing data when using a multilevel modeling approach. For other variables analyzed using rANOVA and rMANOA, multiple imputations will be used. If the assumptions of either Missing Completely at Random (MCAR) or Missing at Random (MAR) are met [87], then these approaches will yield estimates consistent with what would be expected if there were no missing data. Thus, it will allow intention-to-treat analysis (inclusion of all randomized families in the analyses).

## Discussion

This protocol paper outlines the background and design of a randomized controlled trial of *Baby Triple P* for first-time pregnant parents in high-risk populations. It will fill the much-needed gap in the literature as to whether a program that combines parenting, parental wellbeing and the couple relationship is effective in helping high-risk parents ease into the transition to parenthood. This project will employ a rigorous methodology, with multi-domain and multi-informant assessment in order to inform future intervention development and tailoring. Furthermore, by applying an advanced statistical approach we will assist in expanding the knowledge of a variety of intervention outcomes as well as identify aspects for tailoring early parenting interventions in the future. A particular strength of this study will be the recruitment of a high-risk sample that is most likely to benefit from a program, such as Baby Triple P. Moreover, prior intervention studies have largely involved only mothers. This study will encourage both mothers and fathers to attend the intervention together. This will have the added benefit for families, as it will allow for parents to start their parenting journey as a team and research shows that when both parents attend an intervention, the effects are stronger [88, 89]. Further, by evaluating effects for mothers and fathers we will learn whether they benefit from the program differently.

A few limitations of this study should be kept in mind. First, the majority of the assessments are self-report questionnaires, which rely on participant’s honesty, accurate self-reflection and memory of events, as well as their understanding and interpretation of the questions. They also are affected by social biases. However, the inclusion of the observational assessment of responsiveness using the Care Index aims to combat some of the biases of the self-report measures. Another limitation could be the use of a care as usual control group in place of an attention-placebo comparison, as any possible changes after the intervention might be due to the additional support and attention received from the facilitator rather than the specific content of Baby Triple P. However, we have included a strategy checklist to identify if and which of the strategies parents are using, and we can therefore ascertain differences between the groups due to the extent the Baby Triple P strategies are utilized. This would be a good indicator that the intervention itself leads to changes in the outcomes. Furthermore, the use of an attention-placebo control group has it’s own limitations in preventative parenting intervention research, such as unequal drop outs in the control group arm and ethical dilemmas when unsafe parenting behaviors are discussed among parents [90].

Recruitment and enrolment to the study commenced in January 2014 and closed in March 2015. Follow-up data collection was completed in January 2016. During the design phase of this study, the other trials on Baby Triple P discussed earlier were ongoing and results have only become available during the course of this trial. Results will be published in a peer-reviewed journal when analyses are completed. It is expected that participating in a group-based preventative parenting intervention has the potential to help new parents adjust to the transition to parenthood more easily by improving parenting confidence, competence, the parent-infant relationship, parent wellbeing as well as reducing baby behavior problems.

## Abbreviations

BBI: Baby Behaviour Inventory
CAU: Care as usual
CONSORT: Consolidated standards of reporting trials
CWS: Cambridge Worry Scale
EPDS: Edinburgh Postnatal Depression Scale
ES: Effect size
FIML: Full information maximum likelihood analysis
INT: Intervention
MAR: Missing at random
MCAR: Missing completely at random
MSES: Maternal self-efficacy scale
OHQ: Oxford Happiness Questionnaire
PAFAS: Parenting and Family Adjustment Scale
r(M)ANOVA: repeated measures (Multivariate) Analysis of Variance
Triple P: Positive parenting program
